# A chance-constrained program for the allocation of nurses in acute home healthcare

**DOI:** 10.1080/20476965.2025.2467643

**Published:** 2025-02-26

**Authors:** Jedidja Lok-Visser, Hayo Bos, Erwin W. Hans, Gréanne Leeftink

**Affiliations:** Center of Healthcare Operations Improvement and Research, Industrial Engineering & Business Information Systems, University of Twente, Enschede, The Netherlands

**Keywords:** Home healthcare, acute care management, healthcare facility planning, chance-constraints, capacity planning, probabilistic set covering problem

## Abstract

Home healthcare capacity is under great pressure due to demographic developments. Existing literature has exclusively focused on the planning, scheduling, and routing of non-acute care activities. However, similar to other healthcare settings, home healthcare also experiences acute care activities that disrupt operational performance. We study the planning and control of an acute care team for dealing with unplanned and urgent home healthcare activities. Particularly, we focus on determining the number of nurses per care level and their standby locations. The primary aim of this study is to introduce this novel problem, which we define as the acute care team location problem. We formulate this problem as a chance-constrained program. We solve the single location problem to optimality, and the multi-location problem with sample average approximation. The results show that our approach enables decision makers to optimally configure their acute care team, to respond quickly to acute care incidents. From a managerial perspective, our research provides a model that supports tactical capacity planning in HHC organisations and presents a benchmark for acute care management policies.

## 1. Introduction

Home healthcare (HHC) is medical care that is given to a patient at home, rather than in a healthcare facility. Due to ageing populations, demand for HHC services is increasing. Meanwhile, the supply of HHC capacity is decreasing in the Netherlands (Hammink, Mohammadi, & Interreg North Sea 2024), as is also reported in the United States (Scales, 2020). This concerns informal HHC caregivers (Genet et al., 2011; Tarricone & Tsouros, 2008), who are individuals, outside of a professional or formal framework, that have a significant personal relationship with, and who provide a broad range of unpaid assistance to an older person or a person with a chronic or disabling condition (Lilleheie et al., 2020). It also concerns professional nurses, of which there is also a worldwide shortage (McGrath, 2020; Weerdt & Baratta, 2015).

These developments require efficient and effective use of the available capacity by optimising the planning and routing of nurses, which has been a frequently studied topic in recent years (Cissé et al., 2017; DiMascolo et al., 2017; Eenoo et al., 2018; Goodarzian et al., 2023).

The planning and routing of HHC nurses increases in complexity when demand uncertainty is taken into account (Cappanera et al., 2018; Du et al., 2019; Hewitt et al., 2016). Demand uncertainty is amongst others caused by acute incidents, such as fall incidents, and semi-acute incidents, such as changing a colostomy bag. Two emerging initiatives in healthcare, Hospital at Home and Outpatient Parenteral Antimicrobial Therapy, aim to shift hospital care to patients’ homes (Leff et al., 2005; Sadler et al., 2021). This increases the number of acute incidents at home. For instance, incidents with infusion pumps could happen, requiring round-the-clock availability of HHC providers. These acute HHC incidents frequently delay nurses, which affects the highly valued consistent timing of HHC (Woodward et al., 2004). However, the research to date has had only a limited focus on the management of (semi-)acute HHC (Cissé et al., 2017).

Similar to emergency surgeries in hospital operating theatres (Borgman et al., 2021; Duma & Aringhieri, 2019; Van Riet & Demeulemeester, 2015), acute HHC incidents can be dealt with in a generic or a dedicated setting. In the generic setting, acute incidents cause disruptions in the existing schedules of the HHC nurses that deliver regular care (Cappanera et al., 2018; Du et al., 2019), for example, acute incidents may cause delays for planned care, or slack is introduced in the regular HHC nurses’ schedules to accommodate the unforeseen incidents. In the dedicated setting, a dedicated acute care team is formed consisting of nurses that solely respond to the (acute and semi-acute) unplanned care requests. This is similar to emergency teams in hospitals or ambulance teams (Bélanger et al., 2019; Van Oostrum et al., 2008), and both hospitals and HHC organisations have implemented such an acute care team to accommodate their unplanned care requests (Witteveen, 1995).

Where hospital emergency team optimisation has been considered in the literature (Erhard et al., 2018; Paul & MacDonald, 2013), to the best of our knowledge, the optimisation of the configuration of a HHC acute care team has not yet been addressed. The configuration of such a dedicated acute HHC team setting, where nurses are entirely dedicated to acute care incidents during a shift, without responding to elective care, includes various managerial decisions on each level of control (Hulshof et al., 2012). On the strategic level, an organisation has to decide whether such a dedicated setting is preferred over the generic setting and which locations could be possible standby locations. On the tactical level, decision makers determine the number of nurses per qualification level, the standby locations of the nurses, and whether nurses should be relocated. On the offline operational level, the planner determines the routing and scheduling of the nurses, while on the online operational level, nurses can be relocated based on the current state of the system.

In this paper, we address decisions on the tactical level, since a strategic comparison between the dedicated and generic setting can only be made if both settings are sufficiently detailed. For the generic setting organisation, we refer to (Restrepo et al., 2020). The specific decisions refer to the determination of the number of nurses per qualification level and their standby locations such that nurses respond to at least (1 - α)% of the acute incidents (corresponding to a qualification level) in time against minimal costs, where α corresponds to the percentage of acute incidents that the nurses are allowed to not reach in time. Each qualification level can respond to any incidents with a care level up to that qualification level. We define these decisions as the acute care team location problem (ACLP).

For a HHC organisation covering a small region where the nurses can reach all clients within, e.g., 30 minutes, it is reasonable to assume that one standby location is sufficient. This is a special variant of our problem, which we formulate as a single location chance-constrained program (ACLP-1). For HHC organisations covering a wider region, nurses should be distributed over multiple standby locations, to be able to reach all clients within a given response time threshold. For this generalised version, we formulate a chance-constrained program with multiple locations (ACLP-L).

Our contributions are as follows: we introduce a novel optimisation problem in the HHC context, with the objective of addressing a real-world issue. To achieve this, we propose a chance-constrained model that optimises the number of nurses per qualification level in an acute care team and their standby locations. We optimally solve a specific variant of the problem, where only one standby location is available for all nurses. We test this model on a case study in the Netherlands and validate this model with discrete-event simulation (DES). Furthermore, we propose a chance-constrained model that includes multiple standby locations, and which is solved with a sample average approximation (SAA) approach. We test this model on generated instances based on the Solomon instances (Solomon, 1987). We conclude by giving various managerial insights.

The remainder of this paper is organised as follows. Section 2 discusses recent literature on acute HHC incident management and related logistical problems, followed by the problem description and solution approach in Section 3. The experiment design and the outcomes of this approach are presented in Sections 4 and 5. We reflect on our model and solution approach in Section 6.

## 2. Literature

This section provides an overview of HHC literature related to managing acute care incidents and presents a theoretical framework for our research, which describes related research on similar logistical problems in other areas. For reviews on operations research problems in HHC, HHC planning problems, and healthcare facility location problems, we refer to Cissé et al. (2017), Chabouh et al. (2023) and Ahmadi-Javid et al. (2017), respectively.

### 2.1. Acute care management in HHC

We define acute care management in HHC as the organisation of care that is delivered for unscheduled illness and injury (Lotrecchiano et al., 2017), within the home healthcare setting. Examples of this are fall incidents, and semi-acute incidents, such as changing a colostomy bag. This acute care is commonly described as *demand uncertainty* in operations research literature, to distinguish from emergency medical services optimisation. Current research on acute care management in HHC has predominantly focused on limiting the disruption in the schedules of HHC nurses who deal with acute incidents. Chabouh et al. (2023) referred to the inclusion of demand uncertainty as an emerging trend in the HHC literature, but none of the included articles in their review focused on dealing with uncertain demand in a dedicated setting. This section provides some examples of the generic setting for each level of control, to give insight in which objectives are used in related literature.

On the strategic level, Rodriguez-Verjan et al. (2018) determined the locations of HHC centres in a region and their task allocation, for minimal costs while maximizing served demand, solved with a MILP. On the tactical level, research incorporated demand uncertainty over a long time horizon (Hewitt et al., 2016), or revealed uncertain demand one week in advance (Restrepo et al., 2020). Both studies minimised nursing costs, and where Hewitt et al. (2016) focused on minimising travel costs, Restrepo et al. (2020) included continuity of care by assigning nurses to a district, and transitioning them to other districts to respond to unexpected demand. On the operational level Cappanera et al. (2018) and Du et al. (2019) modelled demand uncertainty as unexpected events that disrupt the schedules of HHC nurses. Cappanera and Scutellà (2021) minimised the total turnaround time of the staff and maximised patient satisfaction, based on their preferred time windows and person-oriented consistency. Du et al. (2019) balanced caregiver workload, while ensuring a fast response to all patients. Lastly, Wang et al. (2023) combined multiple levels of control by optimising the staffing decision, how many nurses to hire, with the capacity allocation decision, allocate nurses to service types per day. The demand uncertainty was included both as a known distribution, modelled by stochastic programming, and as an an ambiguity set consisting of multiple distributions, modelled by distributional robust optimisation.

Concluding, current HHC literature has only focused on the generic setting, and the ACLP setting has not been discussed before.

### 2.2. Stochastic demand in related logistical problems

Since existing HHC literature has not focused on managing acute care in a dedicated setting, we review literature on problems similar to the ACLP, which are the call centre staffing problem and the ambulance location problem.

#### 2.2.1. Call centre staffing problem

**Figure 1.**
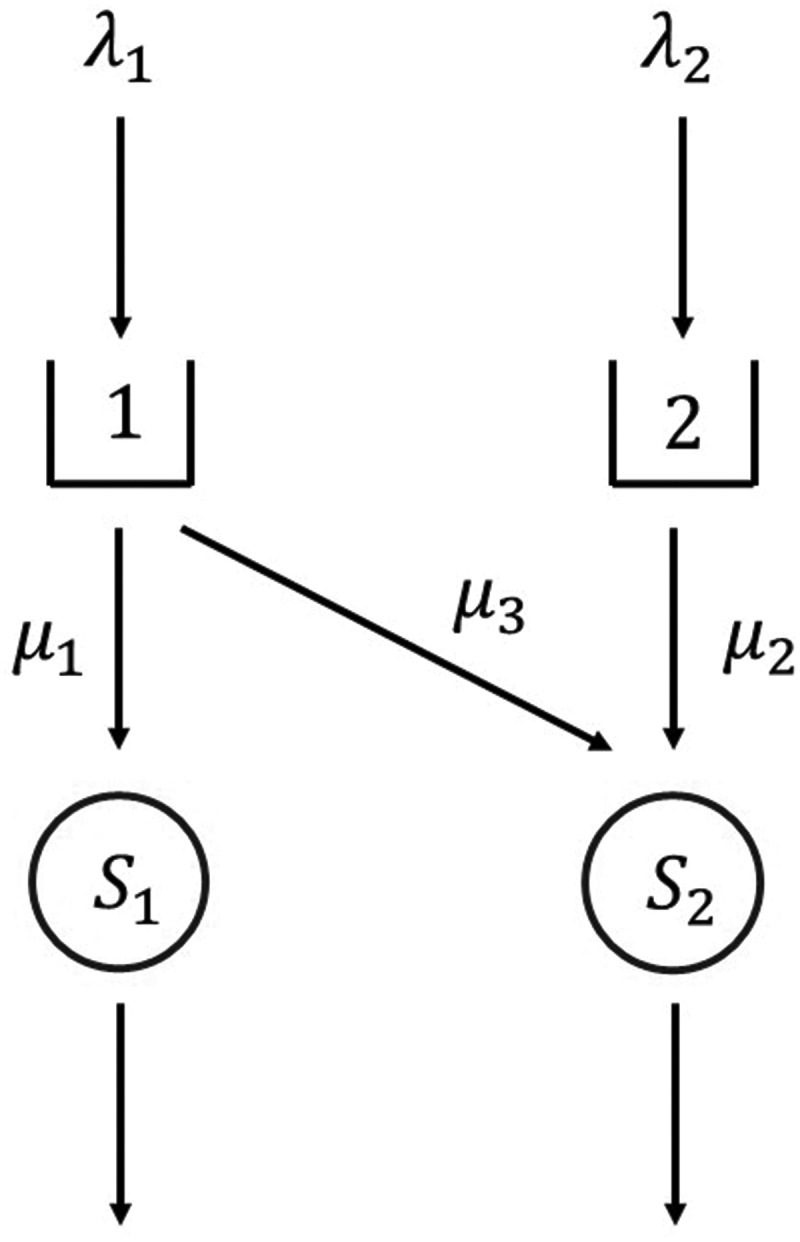
SBR N-design (Garnett & Mandelbaum, 2000).

The special variant of the ACLP with only one standby location is comparable to a call centre staffing problem with uncertainty in the arriving calls. The ACLP can be modelled as a queuing system with multiple types of customers, that require specific skills from the servers. This Markov Decision Process has been called an N-design in skills-based routing as shown in Figure 1 (Gans et al., 2003; Garnett & Mandelbaum, 2000). Bilingual server call centres are an example of this N-design, which have a matrix-geometric solution (Stanford & Grassmann, 2000) under the assumption that there is both a majority and minority language queue. In this M/M/2 system, the call centre staffing problem can be solved asymptotically optimal by a threshold control policy (Bell & Williams, 2001; Gans et al., 2003). In this threshold control policy, S1 always serves type 1 customers, and S2 gives priority to type 2 customers, until the queue of type 1 customers exceeds a certain threshold.

This queuing model is not suitable to find an exact solution for the ACLP. First, the matrix-geometric solution method cannot be applied since the client types queues are often well-balanced. Second, only a limited amount of analytical results for skills-based routing problems exist. Bell and Williams (2001) also did not use an exact approach to solve the N-design staffing problem, they use a simulation approach instead. Third, the ACLP-1 formulation does not focus on service times, but on the concept of maximum concurrent demands, so stochastic optimisation is a more suitable solution approach. An example of a stochastic optimisation approach is provided by Feldman and Mandelbaum (2010), who used stochastic approximation to optimise the staffing of systems with skills-based routing. Simulation-based optimisation is also commonly used to solve these types of problems, of which De Andrade et al. (2023) provided an example.

#### 2.2.2. Ambulance location problem

A second well-studied problem in the literature is the ambulance location problem, which aims to select the standby sites to place emergency vehicles (ambulances), and determines the number of ambulances that should be located at each of them while satisfying a set of constraints (Bélanger et al., 2019). Both the ambulance location problem and the ACLP deal with stochastic demand, have to respond quickly to this demand, and deal with various care levels. The ambulance location problem has been typically modelled as a maximum coverage location problem, which aims to maximise the coverage of the total demand by the ambulances (Ahmadi-Javid et al., 2017). Bélanger et al. (2019) classified the recent ambulance location literature in static location and relocation models. In our HHC context in the Netherlands, online relocation is not yet realistic, since the current information systems do not provide the actual information of all nurses and their locations.

Stochastic and robust location-allocation models could both model the ACLP accurately, since these models minimise the costs under demand satisfaction constraints, considering randomness of demand (Bélanger et al., 2019). These models have been typically described as a two-stage stochastic program, or by a robust optimisation approach. The two-stage stochastic programs available in the ambulance location-allocation literature have various decisions in their first and second stages. Beraldi and Bruni (2009) proposed a two-stage stochastic program that allocates ambulances in the first stage based on uncertain demand that occurs in the second stage. The stochastic constraints were replaced by joint chance-constraints (their probabilistic counterparts) to allow decision makers to evaluate various solutions based on various reliability levels. Khoshgehbari and Mirzapour Al-E-Hashem (2023), Nickel et al. (2016), and Noyan (2010) employed a similar approach. Gago-Carro et al. (2024) and Naoum-Sawaya and Elhedhli (2013) also modelled the ambulance location problem with a two-stage stochastic program, but included relocations and penalties when a call is not served in time. Relocations were determined and minimised in the first stage, where the actual emergency calls response was optimised in the second stage.

Next to stochastic programming, robust optimisation approaches have been proposed for the location-allocation problem. For example, Zhang and Jiang (2014) proposed a robust counterpart of a location-allocation formulation by using the concept of *maximum number of concurrent demands* to estimate the number of emergency vehicles that are needed per station to serve all concurrent demands. They extended their model with a set of chance-constraints to ensure that the number of vehicles per site satisfies the maximum number of concurrent demands assigned to that site. Another example was provided in Wu et al. (2024), who applied distributionally robust optimization models to capture the dynamic and stochastic demand for emergency medical services.

In the HHC context of the ACLP, nurses should respond to at least (1 - α)% of the care requests within a discussed time frame, against minimal costs. Specialised nurses are able to respond to all care requests, while less specialised nurses can only respond to a subset of care requests. To the authors’ knowledge, the current ambulance location theory have not discussed probabilistic covering location models with multiple server types serving different subsets. Mandell (1998) discussed a probabilistic coverage model with two-tiered ambulances, where a less specialised vehicle could arrive earlier than a specialised vehicle, but the specialised vehicle still has to arrive to each request. On the other hand, some studies did include two types of vehicles that could serve different subsets, but in the context of a maximum coverage model (Grannan et al., 2015; McLay, 2009; Yoon et al., 2021) or a minimal costs model without a specific lower bound on the number of missed care requests (Boujemaa et al., 2018).

#### 2.2.3. Conclusion

Concluding, we identify a common and significant problem in HHC, which has not yet been addressed in the literature. Thus, the primary objective of this study is to delineate the ACLP. Furthermore, we introduce an initial approach to solve this novel problem, based on the adjacent area of ambulance location literature. In this approach, we modify a probabilistic coverage model to accommodate multiple server types responding to separate care requests. This leads to a chance-constrained program to solve the ACLP. In the remainder of this paper, we define the ACLP (Section 3.1), formulate our solution approach (Sections 3.2 & 3.3), present the case study and experiment design (Section 4), provide computational results (Section 5), and provide managerial insights for acute care management in HHC (Section 6).

## 3. Problem description and methods

**Figure 2.**
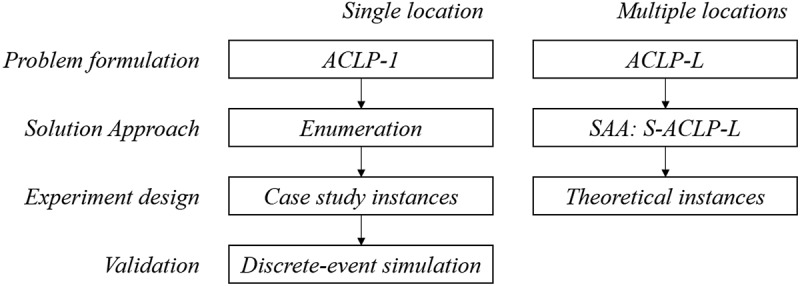
Visualisation of applied methods, experiment design, and validation.

This section introduces the acute care team location problem with single and multiple location(s), and provides a chance-constrained program formulation and solution approach for both these variants. Figure 2 visualises our approach, including the experiment design and validation methods which are presented in Section 4.

### 3.1. ACLP description

Clients in need of care can call the HHC organisation for help at any moment in time (Gammel, 2005). A care coordinator triages the client remotely, and decides whether the client needs an ambulance, an informal caregiver, or an HHC nurse. For the latter, the care coordinator can send a district nurse, who is qualified to assess the healthcare needs of clients and who can provide complex care, or a nurse who is only qualified to give basic care, such as supporting a client after a hygiene incident. Each HHC organisation with an acute care team has to decide on how many district and basic care nurses should be available for unplanned demand, and if relevant, where they should be located to ensure reasonably short response times.

The objective of our problem is to minimise the total hiring and facility costs for responding to a given percentage of the care demand within a given time frame. This objective is based on the perspective of the three main stakeholders of acute HHC organisation, i.e., clients, service provider managers, and nurses. Clients require good quality of service and short response times, managers want to minimise costs, and nurses want to have balanced workloads, autonomy and use a variety of their skills (Carello et al., 2018; Tourangeau et al., 2014).

Both problems, the ACLP-1 that has a single standby location, and the S-ACLP-L, that allocates nurses to multiple locations, are discussed here.

### 3.2. Single location chance-constrained program

Let J be the set of care levels, where the index increases with the complexity of care. Let $c_{j}$ be the costs of hiring a nurse of care level j, j∈J. We assume that a level j nurse can provide care to all clients requiring up to level j care, so a level 1 nurse can only provide care to clients requiring level 1 care, whereas a level |J| nurse can provide care for all patients. Examples of nursing levels j∈J include the licensed practical nurse level, and the registered nurse level. Our goal is to determine the number of required level-j nurses for a specific shift type. A shift is discretised into T consecutive time slots of equal length. We assume deterministic service time of care requests, equal to the length of one time slot. We do not include travel times in this single location problem.

The number of required level-j nurses depends on the random care demand. Let $X_{\mathrm{jt}}$ denote the number of level-j care requests in time slot t. These care requests are unscheduled, and, as is common in emergency department literature, can therefore be modelled as a Poisson process (De Bruin et al., 2010; Green et al., 2006; Kortbeek et al., 2015). We thus assume that $X_{\mathrm{jt}}$ follows a Poisson distribution with parameter $\lambda_{\mathrm{jt}}$. We also assume that all acute care has equal priority. The maximum percentage of time slots in which the scheduled nurses do not have to satisfy all demand of care level j∈J is given by $\alpha_{j}$. The integer valued decision variables $x_{j},j\in J$, represent the number of nurses of care level j that are scheduled in a shift. The objective is to minimise the total hiring costs of the nurses.

The chance-constrained program for the ACLP-1 is given as:

(1) $$\underset{x}{\min}\sum_{j\in J}c_{j}x_{j},$$

subject to

(2) $$P\left(\begin{array}{c} \sum_{\ell =j}^{|J|}x_{\ell }\geq \underset{t}{\max}X_{\mathrm{jt}} \end{array}\right)\geq (1-\alpha_{j}),\;\mathrm{\forall j}\in J,$$

(3) $$x_{j}\in Z_{+},\;\forall j\in J.$$

The objective minimises the nurses’ hiring costs (1). Joint chance-constraints (2) ensure that all demand of care level j in all time slots t should be covered by scheduled nurses in at least $(1-\alpha_{j})\times 100\%$ of the time slots. Similar to the approach of of Zhang and Jiang (2014), the random variable $\max_{t}X_{\mathrm{jt}}$ represents the maximum number of concurrent demand in a time slot t. Constraints (3) are the integrality constraints for the number of nurses to schedule of each care level.

Since the ACLP-1 only depends on the maximum of the random demand vector over all the time slots per shift, we solve this program by enumeration to find the minimal cost configuration given $\alpha_{j}$ for all j∈J. This solution approach allows small home healthcare organisations to implement the model in easily available tools. We solve the ACLP-1 through enumeration by finding the smallest (cost-weighted) integers $x_{j}$ for all j∈J for which we have that

$$P\left(\underset{t}{\max}\left\{\sum_{\ell =j}^{|J|}X_{\mathrm{\ell t}}\right\}\leq \sum_{\ell =j}^{|J|}x_{\ell }\right)=$$

(4) $$\frac{\prod_{t=1}^{T}\;\sum_{j=1}^{\sum_{\ell =j}^{|J|}x_{\ell }}\;{\left(\sum_{\ell =j}^{|J|}\lambda_{\mathrm{\ell t}}\right)}^{j}\exp\left(\sum_{\ell =j}^{|J|}\lambda_{\mathrm{\ell t}}\right)}{j!}\leq \left(1-\frac{\sum_{\ell =j}^{|J|}\;\sum_{t=1}^{T}\lambda_{\mathrm{\ell t}}\alpha_{\ell }}{\sum_{\ell =j}^{|J|}\;\sum_{t=1}^{T}\;}\lambda_{\mathrm{\ell t}}\right),$$

(5) $$x_{j}\geq \sum_{\ell =j}^{|J|}x_{\ell }-x_{j+1},\;j\in \{1,2,\ldots ,|J|-1\},$$

where $\lambda_{\mathrm{jt}}$ denotes the client arrival in time slot t of care level j, and calculate the demand weighted average of $\sum_{\ell =j}^{|J|}\alpha_{\ell }$ for each care level j. Since Constraint (4) determines the minimal number of nurses that is required to respond to all the requests of care level ℓ to |J|, Constraint (5) calculates the actual number of nurses that is required per type.

### 3.3. Chance-constrained program with multiple locations

To extend the ACLP-1 to multiple standby locations for nurses to reach the clients within a preset response time in a larger region, we consider K potential standby locations. To decide which standby locations to use, we need information about the locations of the acute care demand. Let I denote the set of potential acute care demand locations. Let the random variable $a_{\mathrm{ijt}}$ denote the acute demand, which equals 1 if a client at location i, i∈I, requires level-j care, j∈J, at time t, t∈T, and 0 otherwise. Usage of a standby location incurs fixed costs $f_{k},k\in K$. Furthermore, for the binary parameter $r_{\mathrm{ik}}$, $r_{\mathrm{ik}}=1$ indicates that a client at location i∈I can be reached from standby location k∈K within a given response time threshold, and $r_{\mathrm{ik}}=0$ otherwise. Let binary decision variable $y_{k}=1$ if location k∈K is opened, and $y_{k}=0$ otherwise. The auxiliary binary variable $z_{\mathrm{ijkt}}$ links the demand of a client at location i∈I at time t∈T to a nurse of level j∈J from standby location k∈K, and let M be a suitably large number. The objective is to minimise the total hiring costs for nurses as well as the total costs for the standby locations that are opened.

We define the ACLP-L as:

(6) $$\underset{x,y}{\min}\sum_{j\in J}\sum_{k\in K}c_{j}x_{\mathrm{jk}}+\sum_{k\in K}f_{k}y_{k},$$

subject to

(7) $$\sum_{\ell =j}^{|J|}\sum_{k\in K}r_{\mathrm{ik}}z_{i\ell \mathrm{kt}}\geq a_{\mathrm{ijt}}\;\mathrm{\forall i}\in I,t\in T,j\in J,$$

(8) $$P\left(\begin{array}{c} x_{\mathrm{jk}}\geq \underset{t}{\max}\left\{\sum_{i\in I}^{\;}z_{\mathrm{ijkt}}\right\} \end{array}\right)\geq (1-\alpha_{j}),\;\mathrm{\forall j}\in J,$$

(9) $$My_{k}\geq x_{\mathrm{jk}},\;\mathrm{\forall j}\in J,k\in K,$$

(10) $$x_{\mathrm{jk}}\in Z_{+},y_{k}\in B,z_{\mathrm{ijkt}}\in B,\;\forall i\in I,j\in J,k\in K,t\in T.$$

We minimise the total nurse hiring costs and location opening costs (6). Constraints (7) link the client demand to a standby location. Joint chance-constraints (8) ensure that in at least (1 - $\alpha_{j})\times 100\%$ of the time slots, the clients of care level j are covered by a nurse of an appropriate care level that can reach them within the given response time threshold. Constraints (9) ensure to open a standby location if nurses are scheduled at that location, with $M\geq \max_{j,t}\left\{\sum_{i\in I}a_{\mathrm{ijt}}\right\}$. Constraints (10) are sign constraints for the decision and auxiliary variables.

To solve the ACLP-L, we use sample average approximation (SAA) by introducing a big-M constraint as proposed by Pagnoncelli et al. (2009). We consider the set of S scenarios. Let binary variable $\gamma_{\mathrm{js}}$ equal 1 if the demand of the type j∈J clients is not fully covered within the given response time threshold in scenario s∈S, and 0 otherwise. Parameters $M_{1}$ and $M_{2}$ are suitably large numbers.

The scenario formulation of the ACLP-L (S-ACLP-L) is now written as:

(11) $$\underset{x,y}{\min}\sum_{j\in J}\sum_{k\in K}c_{j}x_{\mathrm{jk}}+\sum_{k\in K}f_{k}y_{k},$$

subject to

(12) $$\sum_{\ell =j}^{|J|}\sum_{k\in K}r_{\mathrm{ik}}z_{i\ell \mathrm{kts}}\geq a_{\mathrm{ijts}},\;\mathrm{\forall i}\in I,t\in T,s\in S,$$

(13) $$x_{\mathrm{jk}}+M_{1}\gamma_{\mathrm{js}}\geq \sum_{i\in I}z_{\mathrm{ijkts}},\;\mathrm{\forall j}\in J,k\in K,t\in T,s\in S,$$

(14) $$\sum_{s\in S}\gamma_{\mathrm{js}}\leq |S|\alpha_{j},\;\mathrm{\forall j}\in J,$$

(15) $$M_{2}y_{k}\geq x_{\mathrm{jk}},\;\mathrm{\forall j}\in J,k\in K,$$

(16) $$x_{j}\geq 0,\;\mathrm{\forall j}\in J,$$

(17) $$x_{j}\in Z,y_{k}\in B,z_{\mathrm{ijkts}}\in B,\gamma_{\mathrm{js}}\in B\;\forall i\in I,j\in J,k\in K,t\in T,s\in S.$$

This model is an extended form of model (6)-(10). The auxiliary variables and demand vectors are extended with scenario s in model (11)-(17), with $M_{1},M_{2}\geq \max_{j,t,s}\left\{\sum_{i\in I}\;a_{\mathrm{ijts}}\right\}$. Constraints (13) and (14) ensure that the percentage of scenarios in which the demand coverage constraint of care level j∈J is violated, never exceeds $\alpha_{j}$.

## 4. Experiment design

This section describes the experiment setup to test the performance of the proposed solution approaches for the ACLP-1 and ACLP-L. We introduce the real-life and generated problem instances and experiment settings for both problem formulations in Section 4.1 and 4.2 respectively. To validate the outcomes of the real-life instances, we developed a discrete-event simulation (DES) model, as discussed in Section 4.1.2.

### 4.1. ACLP-1

The analytical approach for the ACLP-1, where the nurses have one standby location, is tested on real-life instances derived from a Dutch HHC organisation, and validated with a DES model.

#### 4.1.1. Case study

To show the applicability of our model in practice, we performed a case study in an HHC organisation in the Netherlands. This organisation recently introduced the acute care team approach. The working area of the HHC organisation is sufficiently small to reach all clients within their threshold from one central location. We therefore modelled this case study as a ACLP-1. Real life data was obtained from March 2021 – December 2021 on the demand requests of the clients, their service times, and their locations. The organisation distinguishes a week and a weekend shift for the acute care team, where the weekend shift tends to be busier. The night shift was excluded, as this shift is organised regionally with other organisations. In this organisation, $J=\left\{\mathrm{basic}\mathrm{care},\mathrm{complex}\mathrm{care}\right\}$. The remaining input parameters are given in Table 1, where $\lambda_{j}$ is the average number of calls of clients with care level j∈J per day. Note that for $\lambda_{j}$, we excluded telephone consultations, and we assumed that the care requests were equally spread over the day. The costs per day were derived from hourly wages discussed in the collective agreements of the Dutch nurse union, as displayed in Table 2 (Sociaal Overleg Verpleeg-, en Verzorgingshuizen en Thuiszorg, 2021).

**Table 1.** Data case study.

$\lambda_{\mathrm{basic}}$ (week)	3.0
$\lambda_{\mathrm{complex}}$ (week)	4.8
$\lambda_{\mathrm{basic}}$ (weekend)	3.3
$\lambda_{\mathrm{complex}}$ (weekend)	5.9
Number of client locations	921
Number of time slots	16
Length of time slot (min)	30

**Table 2.** Costs per day per nurse level given the collective union agreements of the Dutch Nurse Union.

Nurse level 1	€ 137.12
Nurse level 2	€ 173.89

The case study instances were solved by enumeration of (4) and (5), to determine the number of nurses needed per care level both for the week and the weekend shift. The experiment design is a full factorial design of $\alpha_{j}\in \{0.01,0.05,0.1\},\forall j\in J$, resulting in a total of 18 ACLP-1 experiments.

#### 4.1.2. Validation by discrete-event simulation

To validate the results of the ACLP-1 formulation, we developed a discrete-event simulation (DES) model, based on our case study. The goal of this simulation is to validate if assumptions and simplifications in the mathematical modelling of the ACLP, predominately related to service and travel times, and telephone consultations, represent a realistic system.

In this simulation, clients with various urgency levels arrive according to a Poisson distribution, and are served at their location or by telephone by one of the urgent care team nurses. We include random service and travel times, based on empirical distributions obtained from the input data. In addition to basic and complex care, the simulation model also accounted for acute and semi-acute care. We consider a 1:1 ratio for acute and semi-acute clients, based on expert opinion. Acute care always has priority over the other care types, so these clients are visited first-come, first-served (FCFS). For semi-acute care a priority rule is needed to determine which nurse type should respond to a basic care client, and if a district nurse should give priority to a basic or a complex care client. In practice, priority is given according to FCFS, but often there is communication between nurses to check if a basic care nurse is closer and/or almost finished. To simulate the real-life practice of prioritizing clients, we tested two common priority rules: FCFS and the threshold control policy (Gans et al., 2003). In the threshold control policy, the district nurse gives priority to complex care clients, until the queue of basic care clients exceeds a given threshold. Note that in both policies, client pre-emption is not allowed.

Appendix A presents the conceptual model of the DES. Each run consisted of 700 days based on a threshold for statistical significance of 5% and a relative error of 5% (Law, 2015), and each experiment consisted of 20 runs. Output is presented using 95%-confidence intervals of the KPIs percentage of basic care clients served by district nurses; average waiting time for basic and complex care clients; and percentage of clients of basic and complex care that waiting longer than the threshold, respectively.

For each validation experiment of the ACLP-1, we tested the FCFS priority rule and the threshold priority rule, where the latter rule only was tested if there are basic care nurses scheduled. All threshold priority rule experiments were based on a threshold value of 1 for the queue of basic care clients (additional experiments showed that increasing this threshold value gave similar results, and a queue length of 1 is reasonable given the small number of expected clients per day).

### 4.2. S-ACLP-L

**Figure 3.**
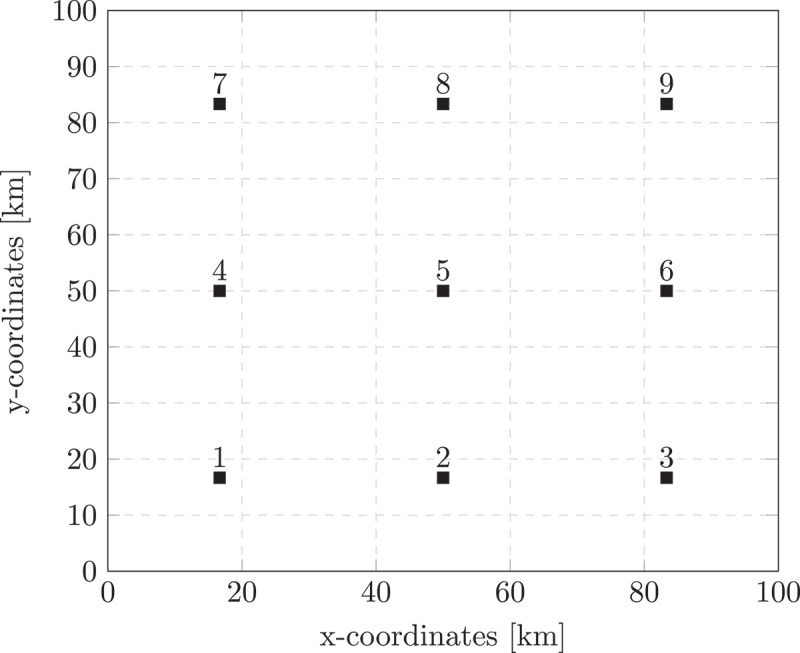
Standby locations on 100×100 km${\;}^{2}$ grid.

For the S-ACLP-L, we generated 100 scenarios as follows. First, we sampled a number of clients per care level per time slot from a Poisson $\left(\frac{\lambda_{j}}{T}\right)$ distribution, as we assumed that demand is equally spread over the day and thinning a Poisson process leads to independent Poisson processes. Then, we assigned demand randomly to client locations, assuming that clients at all locations are equally likely to have an acute incident. These client locations were derived from the customer locations of the Solomon instances (Solomon, 1987). We defined the locations of the Solomon instances on a 100 × 100 km${\;}^{2}$ grid, with nine evenly distributed standby locations as depicted in Figure 3. Clients should be reached within 30 minutes (or 38 kilometres based on the average speed in the Netherlands (Bakker, 2019)) by a nurse from a standby location, similar to the case study organisation. The nursing costs were equal to the costs in the case study instances (see Table 2) and the costs for opening a standby location were arbitrarily set to €100 per day, such that they are in proportion with the nurse costs.

For each instance we assumed a time-independent arrival rate as shown in Table 3. The ACLP-1 and S-ACLP-L are easily extended with time-dependency to deal with peaks in demand during the day or due to seasonality effects. We tested the S-ACLP-L, both for balanced demand and for unbalanced demand, to analyse its effect on the ratio of district nurses to basic care nurses. To analyse the effect of the geographical spread of demand locations, we experimented with random, clustered and random-clustered client locations. For these locations, we used the instance sets that Solomon (1987) developed for the well-known vehicle routing problem with time windows. We used the sets *R101*, *C101* and *RC101* for the random, clustered and random-clustered client location spread, respectively. We considered a full factorial design with all input parameters as shown in Table 3, which led to 54 experiments. We also compared the outcomes with the ACLP-1 approach outcomes for the same configurations without standby locations, to further analyse the effect of the geographical spread of these locations.

**Table 3.** Experiment configurations S-ACLP-L.

Problem parameter	Values considered
Client locations	R101, C101, RC101
Demand arrivals [basic, complex]	[16, 8], [12,12]
$\alpha_{\mathrm{basic}}$	0.1, 0.05, 0.01
$\alpha_{\mathrm{complex}}$	0.1, 0.05, 0.01

We generated 100 scenarios for each S-ACLP-L experiment, since this is the minimum number of scenarios to distinguish between an $\alpha_{j}$ of 0.01 and 0, while the run time is still acceptable.

## 5. Results

This section provides the computational results from the ACLP-1 case study experiments solved with the exact approach (Section 5.1), and the S-ACLP-L experiments solved with SAA, based on the generated instances (Section 5.2). Both models were implemented in Python and the S-ACLP-L experiments were solved with Gurobi version 9.1.1 (Gurobi Optimization, 2022), on a computer equipped with an Intel 2.60 GHz CPU and 16 GB of RAM. The discrete-event simulation model was implemented in Tecnomatix Plant Simulation Software, version 16.1 (Filo et al., 2013), on the same computer.

### 5.1. ACLP-1 results and validation

Table 4 shows the outcomes of the ACLP-1 experiments. From these results, we observe that a basic care nurse was not often included in the configuration of the acute care team. If the basic care nurse was included in a solution, this was often the case for experiments where $\alpha_{\mathrm{basic}}$ is small, whereas $\alpha_{\mathrm{complex}}$ is relatively larger, since an additional nurse was needed to ensure a small $\alpha_{\mathrm{basic}}$, but this did not have to be a district nurses, because of the not too tight $\alpha_{\mathrm{complex}}$.

**Table 4.** ACLP-1 case study results, for each combination of $\alpha_{\mathrm{basic}}$ and $\alpha_{\mathrm{complex}}$ it gives the required nurses in the following order: (basic care during week, complex care during week), (basic care during weekend, complex care during weekend).

$\alpha_{\mathrm{basic}}$\$\alpha_{\mathrm{complex}}$	0.1	0.05	0.01
0.1	(0, 2), (0, 2)	(0, 2), (1, 2)	(0, 3), (0, 3)
0.05	(0, 2), (1, 2)	(1, 2), (1, 2)	(0, 3), (0, 3)
0.01	(0, 2), (1, 2)	(1, 2), (1, 2)	(0, 3), (0, 3)

Table 5 presents the outcomes of the DES, evaluating each of the case study nurse configuration outcomes. This evaluation showed that the percentage of clients that wait longer than the threshold time was always within the required percentage of uncovered demand ($\alpha_{\mathrm{basic}}$ and $\alpha_{\mathrm{complex}}$).

**Table 5.** DES ACLP-1 results.

Demand arrival rates	Nurse configuration	Priority rule	% Basic by district nurses	Avg. WT basic (min)	Avg. WT complex (min)	% Too long WT basic	% Too long WT complex	Total costs per day
Week	(0,2)	FCFS	[41.55; 42.13]	[7.69; 7.80]	[9.29; 9.43]	[1.66; 1.93]	[1.84; 2.05]	€ 347.58
Week	(1,2)	Threshold	[3.19; 3.43]	[8.10; 8.23]	[8.82; 8.94]	[2.51; 2.83]	[1.03; 1.21]	€ 484.70
Week	(1,2)	FCFS	[5.44; 5.73]	[6.77; 6.84]	[8.76; 8.87]	[0.12; 0.21]	[0.89; 1.05]	€ 484.70
Week	(0,3)	FCFS	[41.55; 42.14]	[6.79; 6.86]	[8.37; 8.46]	[0.15; 0.24]	[0.16; 0.22]	€ 521.37
Weekend	(0,2)	FCFS	[40.00; 40.49]	[7.95; 8.13]	[9.91; 10.03]	[2.74; 2.94]	[2.98; 3.20]	€ 347.58
Weekend	(1,2)	Threshold	[3.57; 3.77]	[8.02; 8.19]	[9.14; 9.26]	[3.02; 3.29]	[1.60; 1.70]	€ 484.70
Weekend	(1,2)	FCFS	[5.95; 6.17]	[6.44; 6.56]	[9.08; 9.19]	[0.18; 0.24]	[1.47; 1.58]	€ 484.70
Weekend	(0,3)	FCFS	[40.00; 40.49]	[6.49; 6.61]	[8.47; 8.58]	[0.24; 0.32]	[0.30; 0.37]	€ 521.37

We also conclude that the FCFS priority rule was preferable over the threshold control policy. The threshold control policy resulted in more waiting time for basic care clients, and more clients that had to wait longer than 30 minutes compared to FCFS, while the waiting time for complex care clients did not improve because of the threshold policy. Finally, we conclude that from a nurse perspective, it was beneficial to also include basic care nurses in the acute care team in our case study HHC organisation. As Table 5 shows, the percentage of clients that require basic care and were visited by a district nurse was relatively small. This means that district nurses experienced more job satisfaction because of the complex clients, while basic care nurses experienced more responsibility in their jobs in the acute care team (Tourangeau et al., 2014).

### 5.2. S-ACLP-L results

Table 6 presents the outcomes of all experiments for the S-ACLP-L and ACLP-1. Each experiment shows the number of nurses per education level that were located at each standby location and the corresponding costs, for a specific geographical spread of the clients, a client demand and α‘s. All experiments were solved to optimality within 1000 seconds. The experiments with clustered locations, unbalanced demand and $\alpha_{j}$ = 0.1 took the longest solving time, as their solution space is relatively large, since 10% of the scenarios does not require demand satisfaction.

**Table 6.** Theoretical instances results, with (number of basic care nurses, number of district nurses) per standby location for the S-ACLP-L and ACLP-1. Opened standby locations are grey shaded.

				Standby Locations												
Exp. nr.	Client Locs	[$\lambda_{1}$, $\lambda_{2}$]	[$\alpha_{1}$, $\alpha_{2}$]	1	2	3	4	5	6	7	8	9	Total	ACLP-1	Costs (€)	CPU time (s)
1	R101	[16, 8]	[0.1, 0,01]	(0,1)	(0,1)	(0,0)	(0,1)	(0,1)	(0,0)	(0,0)	(0,0)	(0,0)	(0,4)	(1,3)	1095.16	281
2	R101	[16, 8]	[0.1,0.05]	(0,1)	(0,1)	(0,0)	(0,1)	(0,1)	(0,0)	(0,0)	(0,0)	(0,0)	(0,4)	(1,3)	1095.16	263
3	R101	[16, 8]	[0.1,0.1]	(0,1)	(0,1)	(0,0)	(0,1)	(0,1)	(0,0)	(0,0)	(0,0)	(0,0)	(0,4)	(2,2)	1095.16	258
4	R101	[16, 8]	[0.05,0.01]	(0,1)	(0,1)	(0,0)	(0,1)	(0,1)	(0,0)	(0,0)	(0,0)	(0,0)	(0,4)	(1,3)	1095.16	213
5	R101	[16, 8]	[0.05,0.1]	(0,1)	(0,1)	(0,0)	(0,1)	(0,1)	(0,0)	(0,0)	(0,0)	(0,0)	(0,4)	(2,2)	1095.16	212
6	R101	[16, 8]	[0.05,0.05]	(0,1)	(0,1)	(0,0)	(0,1)	(0,1)	(0,0)	(0,0)	(0,0)	(0,0)	(0,4)	(1,3)	1095.16	208
7	R101	[16, 8]	[0.01,0.1]	(0,1)	(0,1)	(0,0)	(1,1)	(0,1)	(0,0)	(0,0)	(0,0)	(0,0)	(1,4)	(2,2)	1232.28	205
8	R101	[16, 8]	[0.01,0.05]	(0,1)	(0,1)	(0,0)	(1,1)	(0,1)	(0,0)	(0,0)	(0,0)	(0,0)	(1,4)	(2,3)	1232.28	202
9	R101	[16, 8]	[0.01,0.01]	(0,1)	(0,1)	(0,0)	(1,1)	(0,1)	(0,0)	(0,0)	(0,0)	(0,0)	(1,4)	(2,3)	1232.28	198
10	R101	[12, 12]	[0.1,0.01]	(0,1)	(0,1)	(0,0)	(0,1)	(0,1)	(0,0)	(0,0)	(0,0)	(0,0)	(0,4)	(0,4)	1095.16	220
11	R101	[12, 12]	[0.05,0.1]	(0,1)	(0,1)	(0,0)	(0,1)	(0,1)	(0,0)	(0,0)	(0,0)	(0,0)	(0,4)	(1,3)	1095.16	218
12	R101	[12, 12]	[0.05,0.01]	(0,1)	(0,1)	(0,0)	(0,1)	(0,1)	(0,0)	(0,0)	(0,0)	(0,0)	(0,4)	(1,4)	1095.16	214
13	R101	[12, 12]	[0.1,0.05]	(0,1)	(0,1)	(0,0)	(0,1)	(0,1)	(0,0)	(0,0)	(0,0)	(0,0)	(0,4)	(1,3)	1095.16	212
14	R101	[12, 12]	[0.05,0.05]	(0,1)	(0,1)	(0,0)	(0,1)	(0,1)	(0,0)	(0,0)	(0,0)	(0,0)	(0,4)	(1,3)	1095.16	212
15	R101	[12, 12]	[0.1,0.1]	(0,1)	(0,1)	(0,0)	(0,1)	(0,1)	(0,0)	(0,0)	(0,0)	(0,0)	(0,4)	(1,3)	1095.16	209
16	R101	[12, 12]	[0.01,0.01]	(0,1)	(1,1)	(0,0)	(0,1)	(1,1)	(0,0)	(0,0)	(0,0)	(0,0)	(2,4)	(1,4)	1369.40	206
17	R101	[12, 12]	[0.01,0.05]	(0,1)	(1,1)	(0,0)	(0,1)	(1,1)	(0,0)	(0,0)	(0,0)	(0,0)	(2,4)	(2,3)	1369.40	206
18	R101	[12, 12]	[0.01,0.1]	(0,1)	(1,1)	(0,0)	(0,1)	(1,1)	(0,0)	(0,0)	(0,0)	(0,0)	(2,4)	(1,3)	1369.40	204
19	C101	[16, 8]	[0.1,0.01]	(0,0)	(0,1)	(0,0)	(0,2)	(0,0)	(0,1)	(0,0)	(0,1)	(0,0)	(0,5)	(1,3)	1268.95	977
20	C101	[16, 8]	[0.1,0.05]	(0,0)	(0,1)	(0,0)	(0,2)	(0,0)	(0,1)	(0,0)	(0,1)	(0,0)	(0,5)	(1,3)	1268.95	960
21	C101	[16, 8]	[0.1,0.1]	(0,0)	(0,1)	(0,0)	(0,2)	(0,0)	(0,1)	(0,0)	(0,1)	(0,0)	(0,5)	(2,2)	1268.95	926
22	C101	[16, 8]	[0.05,0.1]	(1,1)	(0,0)	(0,0)	(0,0)	(0,1)	(1,1)	(0,0)	(0,1)	(0,0)	(2,4)	(2,2)	1369.40	398
23	C101	[16, 8]	[0.05,0.01]	(1,1)	(0,0)	(0,0)	(0,0)	(0,1)	(1,1)	(0,0)	(0,1)	(0,0)	(2,4)	(1,3)	1369.40	378
24	C101	[16, 8]	[0.05,0.05]	(1,1)	(0,0)	(0,0)	(0,0)	(0,1)	(1,1)	(0,0)	(0,1)	(0,0)	(2,4)	(1,3)	1369.40	376
25	C101	[16, 8]	[0.01,0.01]	(1,1)	(0,0)	(0,0)	(0,0)	(0,1)	(1,1)	(0,0)	(0,2)	(0,0)	(2,5)	(2,3)	1543.19	248
26	C101	[16, 8]	[0.01,0.1]	(1,1)	(0,0)	(0,0)	(0,0)	(0,1)	(1,1)	(0,0)	(0,2)	(0,0)	(2,5)	(2,2)	1543.19	247
27	C101	[16, 8]	[0.01,0.05]	(1,1)	(0,0)	(0,0)	(0,0)	(0,1)	(1,1)	(0,0)	(0,2)	(0,0)	(2,5)	(2,3)	1543.19	245
28	C101	[12, 12]	[0.1,0.01]	(0,1)	(0,0)	(0,0)	(0,1)	(0,0)	(1,1)	(0,0)	(0,1)	(0,0)	(1,4)	(0,4)	1232.28	456
29	C101	[12, 12]	[0.1,0.05]	(0,1)	(0,0)	(0,0)	(0,1)	(0,0)	(1,1)	(0,0)	(0,1)	(0,0)	(1,4)	(1,3)	1232.28	438
30	C101	[12, 12]	[0.1,0.1]	(0,1)	(0,0)	(0,0)	(0,1)	(0,0)	(1,1)	(0,0)	(0,1)	(0,0)	(1,4)	(1,3)	1232.28	415
31	C101	[12, 12]	[0.05,0.1]	(1,1)	(0,0)	(0,0)	(0,0)	(0,1)	(0,1)	(0,0)	(0,2)	(0,0)	(1,5)	(1,3)	1406.07	307
32	C101	[12, 12]	[0.05,0.01]	(1,1)	(0,0)	(0,0)	(0,0)	(0,1)	(0,1)	(0,0)	(0,2)	(0,0)	(1,5)	(1,4)	1406.07	303
33	C101	[12, 12]	[0.05,0.05]	(1,1)	(0,0)	(0,0)	(0,0)	(0,1)	(0,1)	(0,0)	(0,2)	(0,0)	(1,5)	(1,3)	1406.07	298
34	C101	[12, 12]	[0.01,0.1]	(0,2)	(0,0)	(0,0)	(0,0)	(0,1)	(1,1)	(0,0)	(0,2)	(0,0)	(1,6)	(1,3)	1579.86	234
35	C101	[12, 12]	[0.01,0.01]	(0,2)	(0,0)	(0,0)	(0,0)	(0,1)	(1,1)	(0,0)	(0,2)	(0,0)	(1,6)	(1,4)	1579.86	229
36	C101	[12, 12]	[0.01,0.05]	(0,2)	(0,0)	(0,0)	(0,0)	(0,1)	(1,1)	(0,0)	(0,2)	(0,0)	(1,6)	(2,3)	1579.86	227
37	RC101	[16, 8]	[0.1,0.1]	(0,1)	(0,1)	(0,0)	(0,1)	(0,0)	(0,1)	(0,0)	(0,1)	(0,0)	(0,5)	(2,2)	1368.95	412
38	RC101	[16, 8]	[0.1,0.05]	(0,1)	(0,1)	(0,0)	(0,1)	(0,0)	(0,1)	(0,0)	(0,1)	(0,0)	(0,5)	(1,3)	1368.95	412
39	RC101	[16, 8]	[0.1,0.01]	(0,1)	(0,1)	(0,0)	(0,1)	(0,0)	(0,1)	(0,0)	(0,1)	(0,0)	(0,5)	(1,3)	1368.95	408
40	RC101	[16, 8]	[0.05,0.05]	(0,1)	(0,1)	(0,1)	(0,1)	(0,0)	(0,0)	(0,0)	(1,1)	(0,0)	(1,5)	(1,3)	1506.07	320
41	RC101	[16, 8]	[0.05,0.01]	(0,1)	(0,1)	(0,1)	(0,1)	(0,0)	(0,0)	(0,0)	(1,1)	(0,0)	(1,5)	(1,3)	1506.07	319
42	RC101	[16, 8]	[0.05,0.1]	(0,1)	(0,1)	(0,1)	(0,1)	(0,0)	(0,0)	(0,0)	(1,1)	(0,0)	(1,5)	(2,2)	1506.07	313
43	RC101	[16, 8]	[0.01,0.01]	(0,2)	(0,0)	(0,2)	(0,2)	(0,0)	(0,0)	(0,0)	(1,1)	(0,0)	(1,7)	(2,3)	1753.65	219
44	RC101	[16, 8]	[0.01,0.05]	(0,2)	(0,0)	(0,2)	(0,2)	(0,0)	(0,0)	(0,0)	(1,1)	(0,0)	(1,7)	(2,3)	1753.65	209
45	RC101	[16, 8]	[0.01,0.1]	(0,2)	(0,0)	(0,2)	(0,2)	(0,0)	(0,0)	(0,0)	(1,1)	(0,0)	(1,7)	(2,2)	1753.65	209
46	RC101	[12, 12]	[0.1,0.05]	(1,1)	(0,0)	(0,1)	(0,1)	(0,0)	(0,1)	(0,0)	(0,1)	(0,0)	(1,5)	(1,3)	1506.07	476
47	RC101	[12, 12]	[0.1,0.1]	(0,1)	(0,1)	(0,0)	(1,1)	(0,0)	(0,1)	(0,0)	(0,1)	(0,0)	(1,5)	(1,3)	1506.07	421
48	RC101	[12, 12]	[0.1,0.01]	(0,1)	(0,1)	(0,0)	(1,1)	(0,0)	(0,1)	(0,0)	(0,1)	(0,0)	(1,5)	(0,4)	1506.07	419
49	RC101	[12, 12]	[0.05,0.01]	(0,2)	(0,0)	(0,1)	(0,1)	(0,0)	(0,1)	(0,0)	(0,1)	(0,0)	(0,6)	(1,4)	1542.74	225
50	RC101	[12, 12]	[0.05,0.05]	(0,2)	(0,0)	(0,1)	(0,1)	(0,0)	(0,1)	(0,0)	(0,1)	(0,0)	(0,6)	(1,3)	1542.74	224
51	RC101	[12, 12]	[0.05,0.1]	(0,2)	(0,0)	(0,1)	(0,1)	(0,0)	(0,1)	(0,0)	(0,1)	(0,0)	(0,6)	(1,3)	1542.74	222
52	RC101	[12, 12]	[0.01,0.1]	(0,2)	(0,0)	(0,2)	(1,1)	(0,0)	(0,0)	(0,0)	(0,2)	(0,0)	(1,7)	(1,3)	1753.65	210
53	RC101	[12, 12]	[0.01,0.05]	(0,2)	(0,0)	(0,2)	(1,1)	(0,0)	(0,0)	(0,0)	(0,2)	(0,0)	(1,7)	(2,3)	1753.65	208
54	RC101	[12, 12]	[0.01,0.01]	(0,2)	(0,0)	(0,2)	(1,1)	(0,0)	(0,0)	(0,0)	(0,2)	(0,0)	(1,7)	(1,4)	1753.65	203

**Figure 4.**
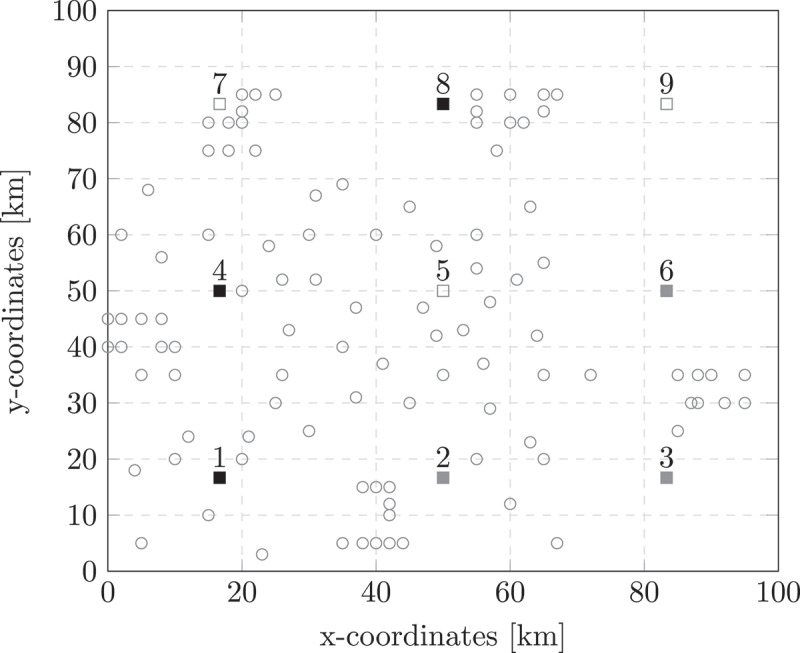
Standby locations selected given a random-clustered geographical spread.

Figure 4 shows the standby locations chosen for a random-clustered geographical spread. The black standby locations are chosen in all experiments, together with a selection of the grey locations. This shows that the S-ACLP-L behaves as a typical probabilistic set covering location problem (Aly & White, 1978).

The solutions of the ACLP-1 for these experiments show the configuration of the team if only one location could be opened. If we compare these outcomes with the S-ACLP-L outcomes, we observe that a wider geographical spread, and thus more standby locations, resulted in more district nurses and fewer basic care nurses to be added to the acute care team. Basic care nurses only seemed beneficial if the number of care requests located close to a standby location was large enough and/or if $\alpha_{\mathrm{basic}}$ was really tight. This makes sense, since the geographical spread of the care requests already requires multiple complex care nurses, which then can also respond to all basic care requests, unless there are many basic care requests or the required basic care coverage is really high.

Table 6 also shows that in our experiments, only $\alpha_{\mathrm{basic}}$ influenced the nurse configurations, where $\alpha_{\mathrm{complex}}$ had no influence on the configurations. For both balanced and unbalanced demand, it appeared that decision makers only have to decide on the percentage of basic care demand that is fulfilled, and can assume that this will fulfil a high percentage of complex care demand as well. As the ACLP-1 results show different behaviour, an explanation could be the geographical spread of the locations which requires at least one district nurse on multiple locations to cover demand. If demand would be unbalanced with more complex care compared to basic care, or if the arrival rate would increase for both care requests, $\alpha_{\mathrm{complex}}$ could have an influence on the nurse configuration.

Based on these experiments, we cannot draw a clear conclusion on the effect of balanced or unbalanced demand. If we compare the experiments with random locations and $\alpha_{\mathrm{basic}}$ = 0.01, more basic care nurses were needed in the balanced demand situation (experiments 16–18, Table 6) than in the experiments with unbalanced demand (experiments 7–9, Table 6). In the balanced demand scenarios, there were more complex care clients that required more district nurses, which could explain why an extra basic care nurse is useful. However, a comparison between the unbalanced (exp. 22–24) and balanced demand (exp. 31–33) experiments with clustered demand and $\alpha_{\mathrm{basic}}$ = 0.05 shows that the unbalanced demand experiments required 2 basic care nurses compared to 1 basic care nurse for the balanced demand. Concluding, the ratio of district nurses to basic care nurses depends on multiple system characteristics.

## 6. Conclusions and discussion

We propose the acute care team location problem (ACLP), which is not discussed yet in the literature. This problem requires a probabilistic set coverage model, with multiple server types responding to separate care requests. For this, we present a chance-constrained program formulation for the single and multiple location ACLP. We formulate initial solution approaches for both variants, and perform experiments based on real-life and theoretical data. These experiments show that the ACLP-1 provides conservative outcomes, and that a FCFS priority rule is preferred over a threshold control policy. Furthermore, from a job satisfaction perspective, adding a basic care nurse to the acute care team is beneficial for our case study HHC organisation. The S-ACLP-L experiments show that basic care nurses are less beneficial if the geographical spread of the clients is wider. We cannot make general conclusions on the effect of the ratio of basic care to complex care clients, but we observe that the S-ACLP-L behaves as a typical probabilistic set covering problem and is therefore useful for HHC organisations.

This research was inspired by a Dutch HHC organisation, who implemented an acute care team in the year leading up to this research. For this organisation, our study led to several benefits and insights. The quantitative results of this research encouraged the decision makers to restructure their data collection process and to start a pilot with basic care nurses in the acute care team, which until then only consisted of district nurses. Our results also substantiated the intuition of the decision makers regarding the FCFS priority rule and including basic care nurses in the team.

For other HHC organisations, our research offers a practical tool to support HHC organisations with their capacity planning decisions on a tactical level. Our models provide an objective reference that organisations can use to benchmark their policies. The planning and scheduling department can easily implement our model based on their own input data, to optimally configure the size, expertise, and standby locations of their acute care team. If a decision should be made on which standby location to use in a ACLP-1 setting, the centre-of-gravity method or the modified gradient procedure can be used, for which we refer to Kuo and White (2004).

Although our study showed that for a small HHC organisation the FCFS policy outperforms the threshold control policy, an organisation could still profit from the intuition behind the threshold control policy, through communication between the district nurses and the basic care nurses. For example, if a basic care nurse is almost finished at a client, the district nurse does not have to respond to a basic care client call.

Note that our solution approaches depend on the assumption that all service times are deterministic and identical, while this is often not the case in practice. Our DES showed for the single location variant of our case study that this assumption is credible, but we suggest that HHC organisations should analyse their historic data to see if this assumption seems viable in practice. Otherwise, the S-ACLP-L could be extended with smaller time slots, where a care demand can occupy multiple time slots, and additional constraints to ensure that the same nurse serves all consecutive time slots. Besides, for organisations with small arrival rates, researchers should be extra careful with the Poisson distribution assumption. For example, the true arrival distribution could be more similar to an exponential distribution, where the Poisson distribution would underestimate the hours with limited arrivals, which should then be taken into account in the interpretation of the results.

As shown in Section 5, accepting a higher percentage of scenarios that do not require demand satisfaction can lead to large solution spaces. If required, planners can either increase the solution time, or decrease the solution space by adding a constraint that the objective function cannot increase the objective function value of a similar experiment with a tighter chance-constraint. The theoretical instances experimented with in this study are rather small, based on realistic proportions for home healthcare organisations in the Netherlands. Larger instances (not reported, but available upon request with the authors) for the S-ACLP-L, supported our insights on opening locations and assigning basic care nurses.

For further research, we propose to compare the acute care team approach with the current common approach in practice, where schedules of nurses are disrupted to respond to acute care incidents. For this research, the Solomon instances could be extended to create a benchmark data set for similar HHC problems. We furthermore propose to extend our S-ACLP-L formulation with client priority weights to distinguish between acute and unplanned care, and to research possible relaxations of the model, since the binary and integer variables reduce the application possibilities. Furthermore, performance indicators related to qualitative measures should be considered, such as job satisfaction (e.g., due to over-qualification or frequent interruptions) and client satisfaction (e.g., due to timely acute care response or delayed regular care). The dynamic acute care team setting, in which acute care team nurses are relocated between different standby locations during their shift, is also an opportunity for further research, for example by applying reinforcement learning.

## Figures and Tables

**Figure A1.**
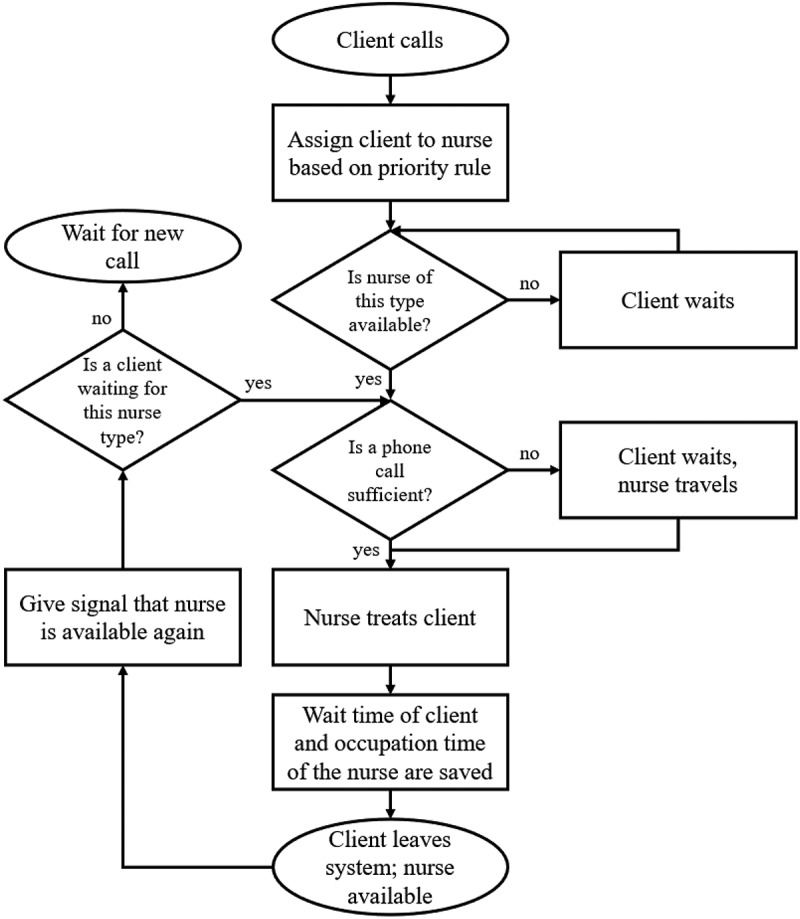
Conceptual model of discrete-event simulation.

## Data Availability

Data is available upon reasonable request with the authors.

## Appendix A

Figure A1 shows the conceptual model of the discrete-event simulation to demonstrate the general client flow through the model.
